# 7-Methoxy-1-{[(*Z*)-3-nitrophenylimino](phenyl)methyl}-2-naphthol

**DOI:** 10.1107/S1600536810039358

**Published:** 2010-10-09

**Authors:** Atsushi Nagasawa, Akiko Okamoto, Noriyuki Yonezawa

**Affiliations:** a Department of Organic and Polymer Materials Chemistry, Tokyo University of Agriculture & Technology, 2-24-16 Naka-machi, Koganei, Tokyo 184-8588, Japan

## Abstract

In the title compound, C_24_H_18_N_2_O_4_, the phenyl and benzene rings are both oriented almost perpendicular to the naphthalene ring system at dihedral angles of 70.97 (5) and 84.64 (5)°. The former rings make a dihedral angle of 87.15 (6)°. The mol­ecule has a *Z* configuration about the C=N bond. In the crystal, mol­ecules are connected by a pair of inter­molecular O—H⋯O hydrogen bonds between the hy­droxy and the nitro group, forming centrosymmetric dimers. Inter­molecular C—H⋯O inter­actions also occur.

## Related literature

For the structures of closely related compounds, see: Hijikata *et al.* (2010 ▶); Watanabe *et al.* (2010 ▶); Mitsui *et al.* (2008 ▶); Nagasawa *et al.* (2010*a* ▶,*b* ▶).
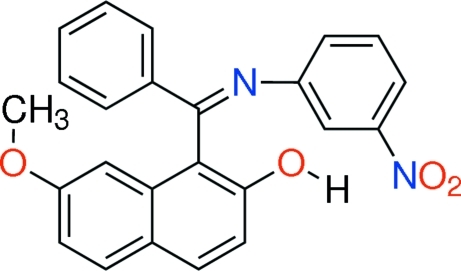


## Experimental

### 

#### Crystal data

C_24_H_18_N_2_O_4_
*M* *_r_* = 398.40
Triclinic, 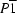

*a* = 9.6709 (10) Å
*b* = 9.8345 (10) Å
*c* = 10.397 (1) Å
α = 88.640 (3)°
β = 89.194 (3)°
γ = 82.126 (3)°
*V* = 979.19 (16) Å^3^
*Z* = 2
Mo *K*α radiation
μ = 0.09 mm^−1^
*T* = 193 K
0.50 × 0.30 × 0.20 mm

#### Data collection

Rigaku R-AXIS RAPID diffractometer
Absorption correction: numerical (*NUMABS*; Higashi, 1999 ▶) *T*_min_ = 0.955, *T*_max_ = 0.982
15901 measured reflections
4475 independent reflections
3923 reflections with *I* > 2σ(*I*)
*R*_int_ = 0.014

#### Refinement

*R*[*F* ^2^ > 2σ(*F* ^2^)] = 0.043
*wR*(*F* ^2^) = 0.156
*S* = 1.13
4475 reflections
277 parameters
1 restraint
H atoms treated by a mixture of independent and constrained refinement
Δρ_max_ = 0.40 e Å^−3^
Δρ_min_ = −0.40 e Å^−3^

### 

Data collection: *PROCESS-AUTO* (Rigaku, 1998 ▶); cell refinement: *PROCESS-AUTO*; data reduction: *CrystalStructure* (Rigaku/MSC, 2004 ▶); program(s) used to solve structure: *SIR2004* (Burla *et al.*, 2005 ▶); program(s) used to refine structure: *SHELXL97* (Sheldrick, 2008 ▶); molecular graphics: *ORTEPIII* (Burnett & Johnson, 1996 ▶); software used to prepare material for publication: *SHELXL97*.

## Supplementary Material

Crystal structure: contains datablocks I, global. DOI: 10.1107/S1600536810039358/pk2274sup1.cif

Structure factors: contains datablocks I. DOI: 10.1107/S1600536810039358/pk2274Isup2.hkl

Additional supplementary materials:  crystallographic information; 3D view; checkCIF report

## Figures and Tables

**Table 1:** Hydrogen-bond geometry (Å, °)

*D*—H⋯*A*	*D*—H	H⋯*A*	*D*⋯*A*	*D*—H⋯*A*
O1—H1⋯O3^i^	0.83 (2)	2.05 (2)	2.8559 (17)	163 (18)
C19—H19⋯O1	0.95	2.56	3.3241 (16)	138

Symmetry code: (i) 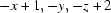.

**Fig. 1.**
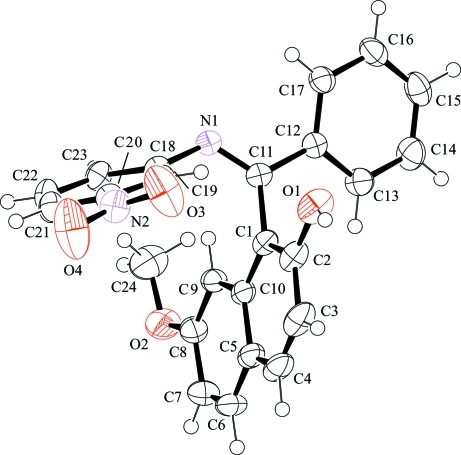
The asymmetric unit of the title compound, showing 50% probability displacement ellipsoids.

**Fig. 2.**
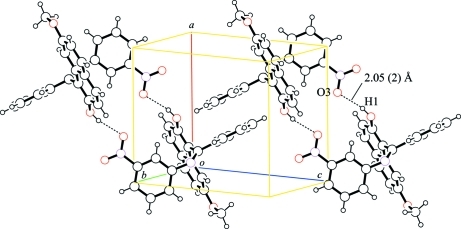
A partial crystal packing diagram of the compound, viewed down the b axis. Intermolecular O—H···O hydrogen bonds are shown as dashed lines.

**Fig. 3.**
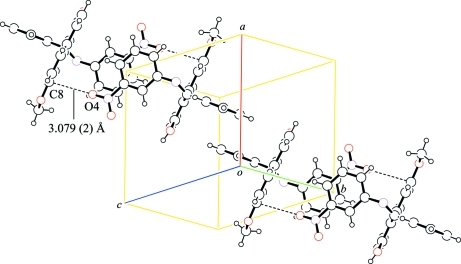
A partial crystal packing diagram of the compound, viewed down the b axis. Intermolecular N≐O···C interactions are shown as dashed lines.

## supplementary crystallographic information

### Comment

Recently, we reported crystal structures of several 1-monoaroylnaphthalene derivatives exemplified by 2-(2,7-dimethoxy-1-naphthoyl)benzoic acid (Hijikata *et al.*, 2010), 2,7-dimethoxy-1-(4-nitrobenzoyl)-naphthalene (Watanabe *et al.*, 2010) and (4-chlorophenyl)(2-hydroxy-7-methoxynaphthalen-1-yl)methanone (Mitsui *et al.*, 2008). Furthermore, we also reported the crystal structure of 1-[(4-chlorophenyl)(phenylimino)methyl]-7-methoxy-2-naphthol-1,4-diazabicyclo[2.2.2]octane (2/1) (Nagasawa *et al.*, 2010*a*) that formed 2:1 comolecular unit of triarylimine and 1,4-diazabicyclo[2.2.2]octane (DABCO). As a part of the continuous study on the molecular structures of this kind of homologous molecules, we have investigated imination reaction of aroylated naphthalene. The title compound was prepared from (2-hydroxy-7-methoxynaphthalen-1-yl)(phenyl)methanone (Nagasawa *et al.*, 2010*b*) and 3-nitroaniline in the presence of TiCl_4_ and DABCO.

An *ORTEPIII* (Burnett & Johnson, 1996) plot is shown in Fig. 1. In the molecule, interplanar angles of the least-squares plane of the benzene ring (C12–C17) attached to nitrogen atom (N1) and benzene ring (C18–C23) attached to carbon atom (C11) of imine moiety against the naphthalene ring (C1–C10) are 70.97 (5) and 84.64 (5)°, respectively. Furthermore, the interplanar angle between two benzene rings is 87.15 (6)°. The molecule has a *Z* configuration for the imine vector.

In the crystal structure, the molecular packing is mainly stabilized by intermolecular hydrogen bond and van der Waals interactions. The intermolecular O—H···O hydrogen bond between the hydorxy and nitro groups on the naphthalene ring and the *N*-aryl group along the *c* axis, is observed [H1···O3 = 2.05 (2) Å] (Fig. 2). The carbon atom in the naphthalene ring interacts with an oxygen atom in the nitro groups [C8···O4 = 3.079 (2) Å] along the *c* axis (Fig. 3). One hydrogen atom on a phenyl group has a close contact with the hydrogen atom on the phenyl group of the next molecule [H16···H17 = 2.27 Å], roughly along the *a* axis.

### Experimental

To a solution of (2-hydroxy-7-methoxynaphthalen-1- yl)(phenyl)methanone (0.2 mmol, 56 mg) in chlorobenzene (1 ml), a mixture of 3-nitroaniline (0.22 mmol, 30 mg), TiCl_4_ (0.33 mmol, 62.4 mg), DABCO (1.32 mmol, 148.0 mg) and chlorobenzene (1 ml) was added by portions at 363 K under nitrogen atmosphere. After the reaction mixture was stirred at 398 K for 1.5 h, the resulting solution was filtered to remove the precipitate. The solvent was removed under reduced pressure to give crude material. The crude material thus obtained was subjected to crystallization from CHCl_3_/hexane to give compind (I) as yellow platelets (m.p. 508.5–509.0 K, yield 28 mg, 35%).

Spectroscopic Data: ^1^H NMR (300 MHz, DMSO-*d_6_*) δ; 10.23, (s, 1H), 7.69–7.60 (m, 6H), 7.49–7.38 (m, 3H), 7.30–7.26 (m, 2H), 6.97 (d, 1H), 6.84 (dd, 1H), 6.54 (d, 1H), 3.66 (s, 3H); ^13^C NMR (75 MHz, DMSO-*d_6_*) 168.5, 158.7, 154.1, 152.8, 148.0, 138.3, 132.5, 131.9, 131.0, 130.5, 130.1, 129.2, 128.7, 126.6, 123.1, 118.7, 115.6, 115.2, 114.8, 114.2, 102.6, 55.6; IR (KBr): 3416, 3073, 1626, 1614, 1511, 1341, 1210; HRMS (*m/z*): [*M* + H]^+^ calcd for C_24_H_19_N_2_O_4_, 399.1345; found, 399.1349.

### Refinement

All the H-atoms could be located in difference Fourier maps. The O—H hydrogen atom was freely refined: O1—H1 = 0.89 (2) Å. The C-bound H-atoms were subsequently refined as riding atoms, with C—H = 0.95 (aromatic) and 0.98 (methyl) Å, and *U_iso_*(H) = 1.2*U_eq_*(C). Rigid bond restraints were applied to the *U*_ij_ values of the naphthalene ring (C6 and C7) [1 restraint with the DELU command in *SHELXL97*].

### Crystal data

**Table:**

C_24_H_18_N_2_O_4_	*Z* = 2
*M**_r_* = 398.40	*F*(000) = 416
Triclinic, *P*1	*D*_x_ = 1.351 Mg m^−^^3^
Hall symbol: -P 1	Melting point = 509.0–508.5 K
*a* = 9.6709 (10) Å	Mo *K*α radiation, λ = 0.71075 Å
*b* = 9.8345 (10) Å	Cell parameters from 11810 reflections
*c* = 10.397 (1) Å	θ = 3.2–27.4°
α = 88.640 (3)°	µ = 0.09 mm^−^^1^
β = 89.194 (3)°	*T* = 193 K
γ = 82.126 (3)°	Platelet, yellow
*V* = 979.19 (16) Å^3^	0.50 × 0.30 × 0.20 mm

### Data collection

**Table:**

Rigaku R-AXIS RAPID diffractometer	4475 independent reflections
Radiation source: rotating anode	3923 reflections with *I* > 2σ(*I*)
graphite	*R*_int_ = 0.014
Detector resolution: 10.00 pixels mm^-1^	θ_max_ = 27.4°, θ_min_ = 3.2°
ω scans	*h* = −12→12
Absorption correction: numerical (*NUMABS*; Higashi, 1999)	*k* = −12→12
*T*_min_ = 0.955, *T*_max_ = 0.982	*l* = −12→13
15901 measured reflections	

### Refinement

**Table:**

Refinement on *F*^2^	Secondary atom site location: difference Fourier map
Least-squares matrix: full	Hydrogen site location: inferred from neighbouring sites
*R*[*F*^2^ > 2σ(*F*^2^)] = 0.043	H atoms treated by a mixture of independent and constrained refinement
*wR*(*F*^2^) = 0.156	*w* = 1/[σ^2^(*F*_o_^2^) + (0.098*P*)^2^ + 0.153*P*] where *P* = (*F*_o_^2^ + 2*F*_c_^2^)/3
*S* = 1.13	(Δ/σ)_max_ < 0.001
4475 reflections	Δρ_max_ = 0.40 e Å^−^^3^
277 parameters	Δρ_min_ = −0.40 e Å^−^^3^
1 restraint	Extinction correction: *SHELXL*, Fc^*^=kFc[1+0.001xFc^2^λ^3^/sin(2θ)]^-1/4^
Primary atom site location: structure-invariant direct methods	Extinction coefficient: 0.039 (6)

### Special details

**Table:**

Geometry. All e.s.d.'s (except the e.s.d. in the dihedral angle between two l.s. planes) are estimated using the full covariance matrix. The cell e.s.d.'s are taken into account individually in the estimation of e.s.d.'s in distances, angles and torsion angles; correlations between e.s.d.'s in cell parameters are only used when they are defined by crystal symmetry. An approximate (isotropic) treatment of cell e.s.d.'s is used for estimating e.s.d.'s involving l.s. planes.
Refinement. Refinement of *F*^2^ against ALL reflections. The weighted *R*-factor *wR* and goodness of fit *S* are based on *F*^2^, conventional *R*-factors *R* are based on *F*, with *F* set to zero for negative *F*^2^. The threshold expression of *F*^2^ > σ(*F*^2^) is used only for calculating *R*-factors(gt) *etc*. and is not relevant to the choice of reflections for refinement. *R*-factors based on *F*^2^ are statistically about twice as large as those based on *F*, and *R*- factors based on ALL data will be even larger.

### Fractional atomic coordinates and isotropic or equivalent isotropic displacement parameters (Å^2^)

**Table:**

	*x*	*y*	*z*	*U*_iso_*/*U*_eq_	
O1	0.51816 (11)	0.18118 (11)	0.75931 (11)	0.0491 (3)	
O2	1.18407 (11)	0.40281 (11)	0.59012 (12)	0.0531 (3)	
O3	0.64352 (12)	−0.1348 (2)	1.01592 (12)	0.0759 (5)	
O4	0.82233 (15)	−0.1964 (2)	1.12868 (14)	0.0907 (6)	
N1	0.81213 (10)	0.03212 (10)	0.59401 (9)	0.0280 (2)	
N2	0.76948 (13)	−0.15267 (13)	1.02938 (11)	0.0411 (3)	
C1	0.73411 (13)	0.24880 (12)	0.70311 (10)	0.0289 (3)	
C2	0.61980 (15)	0.25958 (13)	0.78516 (12)	0.0367 (3)	
C3	0.61168 (17)	0.34798 (16)	0.89160 (13)	0.0469 (4)	
H3	0.5344	0.3522	0.9495	0.056*	
C4	0.71436 (18)	0.42664 (15)	0.91081 (13)	0.0470 (4)	
H4	0.7078	0.4854	0.9825	0.056*	
C5	0.83046 (16)	0.42299 (13)	0.82655 (12)	0.0395 (3)	
C6	0.93661 (19)	0.50761 (15)	0.84159 (16)	0.0516 (4)	
H6	0.9286	0.5711	0.9096	0.062*	
C7	1.04946 (18)	0.50021 (16)	0.76113 (18)	0.0522 (4)	
H7	1.1184	0.5588	0.7727	0.063*	
C8	1.06394 (15)	0.40493 (13)	0.66003 (14)	0.0400 (3)	
C9	0.96183 (13)	0.32336 (12)	0.63937 (12)	0.0314 (3)	
H9	0.9710	0.2620	0.5696	0.038*	
C10	0.84274 (13)	0.33059 (12)	0.72195 (11)	0.0302 (3)	
C11	0.74138 (11)	0.15216 (11)	0.59275 (10)	0.0261 (2)	
C12	0.66388 (11)	0.20074 (12)	0.47405 (11)	0.0273 (3)	
C13	0.63505 (14)	0.34105 (13)	0.44511 (12)	0.0360 (3)	
H13	0.6636	0.4057	0.5021	0.043*	
C14	0.56463 (16)	0.38643 (15)	0.33290 (13)	0.0437 (3)	
H14	0.5462	0.4819	0.3129	0.052*	
C15	0.52155 (14)	0.29251 (17)	0.25059 (13)	0.0427 (3)	
H15	0.4724	0.3237	0.1747	0.051*	
C16	0.54984 (13)	0.15291 (16)	0.27845 (13)	0.0404 (3)	
H16	0.5210	0.0888	0.2211	0.048*	
C17	0.62029 (12)	0.10671 (13)	0.39009 (12)	0.0337 (3)	
H17	0.6388	0.0111	0.4093	0.040*	
C18	0.87584 (12)	−0.02373 (11)	0.70917 (11)	0.0270 (2)	
C19	0.79334 (12)	−0.05397 (12)	0.81420 (11)	0.0297 (3)	
H19	0.6947	−0.0306	0.8122	0.036*	
C20	0.85794 (13)	−0.11863 (12)	0.92129 (11)	0.0311 (3)	
C21	1.00158 (14)	−0.15383 (14)	0.93041 (13)	0.0377 (3)	
H21	1.0431	−0.1963	1.0061	0.045*	
C22	1.08160 (13)	−0.12449 (14)	0.82466 (14)	0.0399 (3)	
H22	1.1801	−0.1482	0.8273	0.048*	
C23	1.02033 (13)	−0.06076 (13)	0.71432 (12)	0.0339 (3)	
H23	1.0771	−0.0425	0.6424	0.041*	
C24	1.20954 (18)	0.30536 (19)	0.4914 (2)	0.0579 (4)	
H24A	1.3023	0.3094	0.4536	0.069*	
H24B	1.1387	0.3261	0.4247	0.069*	
H24C	1.2052	0.2131	0.5276	0.069*	
H1	0.464 (3)	0.185 (3)	0.822 (2)	0.076 (7)*	

### Atomic displacement parameters (Å^2^)

**Table:**

	*U*^11^	*U*^22^	*U*^33^	*U*^12^	*U*^13^	*U*^23^
O1	0.0450 (6)	0.0501 (6)	0.0521 (6)	−0.0085 (5)	0.0258 (5)	−0.0018 (5)
O2	0.0429 (6)	0.0436 (6)	0.0771 (8)	−0.0202 (5)	0.0011 (5)	−0.0073 (5)
O3	0.0408 (6)	0.1428 (14)	0.0451 (7)	−0.0213 (7)	0.0094 (5)	0.0244 (8)
O4	0.0673 (9)	0.1390 (15)	0.0587 (8)	−0.0001 (9)	0.0038 (6)	0.0621 (9)
N1	0.0285 (5)	0.0284 (5)	0.0273 (5)	−0.0052 (4)	0.0036 (3)	−0.0012 (4)
N2	0.0428 (6)	0.0427 (6)	0.0377 (6)	−0.0076 (5)	0.0058 (5)	0.0080 (5)
C1	0.0369 (6)	0.0261 (5)	0.0224 (5)	0.0000 (4)	0.0039 (4)	0.0015 (4)
C2	0.0445 (7)	0.0319 (6)	0.0314 (6)	0.0015 (5)	0.0110 (5)	0.0039 (5)
C3	0.0622 (9)	0.0442 (7)	0.0285 (6)	0.0119 (7)	0.0167 (6)	0.0015 (5)
C4	0.0715 (10)	0.0383 (7)	0.0258 (6)	0.0129 (7)	−0.0007 (6)	−0.0076 (5)
C5	0.0565 (8)	0.0299 (6)	0.0291 (6)	0.0059 (6)	−0.0090 (5)	−0.0040 (5)
C6	0.0715 (10)	0.0347 (7)	0.0476 (8)	0.0000 (7)	−0.0208 (7)	−0.0152 (6)
C7	0.0567 (9)	0.0362 (7)	0.0656 (10)	−0.0102 (6)	−0.0183 (7)	−0.0121 (7)
C8	0.0427 (7)	0.0298 (6)	0.0486 (8)	−0.0076 (5)	−0.0098 (6)	−0.0006 (5)
C9	0.0377 (6)	0.0259 (5)	0.0312 (6)	−0.0058 (5)	−0.0049 (5)	−0.0018 (4)
C10	0.0411 (6)	0.0249 (5)	0.0232 (5)	0.0007 (5)	−0.0048 (4)	0.0014 (4)
C11	0.0263 (5)	0.0278 (5)	0.0251 (5)	−0.0078 (4)	0.0066 (4)	0.0001 (4)
C12	0.0247 (5)	0.0315 (6)	0.0259 (5)	−0.0054 (4)	0.0051 (4)	0.0009 (4)
C13	0.0440 (7)	0.0334 (6)	0.0308 (6)	−0.0067 (5)	−0.0003 (5)	0.0029 (5)
C14	0.0515 (8)	0.0416 (7)	0.0361 (7)	−0.0009 (6)	−0.0021 (6)	0.0098 (5)
C15	0.0349 (6)	0.0617 (9)	0.0299 (6)	−0.0011 (6)	−0.0027 (5)	0.0047 (6)
C16	0.0299 (6)	0.0542 (8)	0.0377 (7)	−0.0068 (6)	−0.0041 (5)	−0.0073 (6)
C17	0.0275 (5)	0.0367 (6)	0.0376 (6)	−0.0065 (5)	−0.0002 (5)	−0.0027 (5)
C18	0.0299 (5)	0.0230 (5)	0.0284 (5)	−0.0040 (4)	0.0024 (4)	−0.0026 (4)
C19	0.0258 (5)	0.0303 (6)	0.0333 (6)	−0.0050 (4)	0.0025 (4)	0.0001 (4)
C20	0.0339 (6)	0.0282 (5)	0.0315 (6)	−0.0060 (5)	0.0043 (4)	0.0019 (4)
C21	0.0365 (6)	0.0346 (6)	0.0404 (7)	−0.0006 (5)	−0.0043 (5)	0.0072 (5)
C22	0.0272 (6)	0.0418 (7)	0.0483 (8)	0.0025 (5)	0.0015 (5)	0.0046 (6)
C23	0.0293 (6)	0.0343 (6)	0.0373 (6)	−0.0019 (5)	0.0079 (5)	0.0003 (5)
C24	0.0444 (8)	0.0529 (9)	0.0798 (12)	−0.0189 (7)	0.0171 (8)	−0.0103 (8)

### Geometric parameters (Å, °)

**Table:**

O1—C2	1.3630 (18)	C11—C12	1.4876 (15)
O1—H1	0.83 (3)	C12—C13	1.3963 (17)
O2—C8	1.3597 (19)	C12—C17	1.3965 (17)
O2—C24	1.417 (2)	C13—C14	1.3923 (18)
O3—N2	1.2162 (17)	C13—H13	0.9500
O4—N2	1.2023 (17)	C14—C15	1.383 (2)
N1—C11	1.2807 (15)	C14—H14	0.9500
N1—C18	1.4184 (14)	C15—C16	1.387 (2)
N2—C20	1.4630 (16)	C15—H15	0.9500
C1—C2	1.3809 (17)	C16—C17	1.3893 (18)
C1—C10	1.4261 (18)	C16—H16	0.9500
C1—C11	1.5020 (15)	C17—H17	0.9500
C2—C3	1.418 (2)	C18—C19	1.3938 (16)
C3—C4	1.359 (2)	C18—C23	1.3963 (16)
C3—H3	0.9500	C19—C20	1.3825 (17)
C4—C5	1.412 (2)	C19—H19	0.9500
C4—H4	0.9500	C20—C21	1.3888 (18)
C5—C6	1.420 (2)	C21—C22	1.3832 (19)
C5—C10	1.4269 (17)	C21—H21	0.9500
C6—C7	1.361 (3)	C22—C23	1.3940 (18)
C6—H6	0.9500	C22—H22	0.9500
C7—C8	1.417 (2)	C23—H23	0.9500
C7—H7	0.9500	C24—H24A	0.9800
C8—C9	1.3770 (17)	C24—H24B	0.9800
C9—C10	1.4217 (18)	C24—H24C	0.9800
C9—H9	0.9500		
			
C2—O1—H1	107.8 (17)	C17—C12—C11	120.44 (10)
C8—O2—C24	117.74 (11)	C14—C13—C12	120.17 (12)
C11—N1—C18	120.16 (10)	C14—C13—H13	119.9
O4—N2—O3	121.85 (12)	C12—C13—H13	119.9
O4—N2—C20	119.64 (12)	C15—C14—C13	119.99 (13)
O3—N2—C20	118.51 (11)	C15—C14—H14	120.0
C2—C1—C10	120.16 (11)	C13—C14—H14	120.0
C2—C1—C11	119.33 (11)	C14—C15—C16	120.26 (12)
C10—C1—C11	120.51 (10)	C14—C15—H15	119.9
O1—C2—C1	116.99 (12)	C16—C15—H15	119.9
O1—C2—C3	122.55 (12)	C15—C16—C17	120.13 (12)
C1—C2—C3	120.45 (13)	C15—C16—H16	119.9
C4—C3—C2	120.05 (13)	C17—C16—H16	119.9
C4—C3—H3	120.0	C16—C17—C12	120.05 (12)
C2—C3—H3	120.0	C16—C17—H17	120.0
C3—C4—C5	121.45 (12)	C12—C17—H17	120.0
C3—C4—H4	119.3	C19—C18—C23	119.36 (11)
C5—C4—H4	119.3	C19—C18—N1	119.97 (10)
C4—C5—C6	122.65 (13)	C23—C18—N1	120.40 (10)
C4—C5—C10	119.09 (13)	C20—C19—C18	118.63 (11)
C6—C5—C10	118.26 (14)	C20—C19—H19	120.7
C7—C6—C5	121.75 (13)	C18—C19—H19	120.7
C7—C6—H6	119.1	C19—C20—C21	123.33 (11)
C5—C6—H6	119.1	C19—C20—N2	117.89 (11)
C6—C7—C8	119.90 (14)	C21—C20—N2	118.78 (11)
C6—C7—H7	120.1	C22—C21—C20	117.21 (12)
C8—C7—H7	120.1	C22—C21—H21	121.4
O2—C8—C9	125.18 (13)	C20—C21—H21	121.4
O2—C8—C7	114.36 (13)	C21—C22—C23	121.21 (11)
C9—C8—C7	120.46 (14)	C21—C22—H22	119.4
C8—C9—C10	120.30 (12)	C23—C22—H22	119.4
C8—C9—H9	119.9	C22—C23—C18	120.24 (11)
C10—C9—H9	119.9	C22—C23—H23	119.9
C9—C10—C1	122.02 (10)	C18—C23—H23	119.9
C9—C10—C5	119.27 (12)	O2—C24—H24A	109.5
C1—C10—C5	118.71 (12)	O2—C24—H24B	109.5
N1—C11—C12	118.34 (10)	H24A—C24—H24B	109.5
N1—C11—C1	123.99 (10)	O2—C24—H24C	109.5
C12—C11—C1	117.66 (9)	H24A—C24—H24C	109.5
C13—C12—C17	119.39 (11)	H24B—C24—H24C	109.5
C13—C12—C11	120.16 (10)		
			
C10—C1—C2—O1	178.22 (10)	C10—C1—C11—N1	81.52 (14)
C11—C1—C2—O1	−0.66 (17)	C2—C1—C11—C12	81.77 (13)
C10—C1—C2—C3	−2.39 (19)	C10—C1—C11—C12	−97.11 (12)
C11—C1—C2—C3	178.72 (11)	N1—C11—C12—C13	−153.41 (11)
O1—C2—C3—C4	−178.23 (13)	C1—C11—C12—C13	25.30 (15)
C1—C2—C3—C4	2.4 (2)	N1—C11—C12—C17	26.07 (15)
C2—C3—C4—C5	0.1 (2)	C1—C11—C12—C17	−155.22 (11)
C3—C4—C5—C6	177.66 (13)	C17—C12—C13—C14	−0.60 (18)
C3—C4—C5—C10	−2.5 (2)	C11—C12—C13—C14	178.89 (11)
C4—C5—C6—C7	178.39 (14)	C12—C13—C14—C15	0.8 (2)
C10—C5—C6—C7	−1.4 (2)	C13—C14—C15—C16	−0.9 (2)
C5—C6—C7—C8	−0.8 (2)	C14—C15—C16—C17	0.7 (2)
C24—O2—C8—C9	−2.7 (2)	C15—C16—C17—C12	−0.54 (19)
C24—O2—C8—C7	176.88 (14)	C13—C12—C17—C16	0.47 (18)
C6—C7—C8—O2	−177.06 (14)	C11—C12—C17—C16	−179.01 (10)
C6—C7—C8—C9	2.6 (2)	C11—N1—C18—C19	65.82 (14)
O2—C8—C9—C10	177.50 (12)	C11—N1—C18—C23	−120.21 (12)
C7—C8—C9—C10	−2.1 (2)	C23—C18—C19—C20	0.75 (17)
C8—C9—C10—C1	179.41 (11)	N1—C18—C19—C20	174.77 (10)
C8—C9—C10—C5	−0.16 (18)	C18—C19—C20—C21	0.78 (19)
C2—C1—C10—C9	−179.62 (11)	C18—C19—C20—N2	−178.51 (10)
C11—C1—C10—C9	−0.75 (17)	O4—N2—C20—C19	−172.65 (16)
C2—C1—C10—C5	−0.05 (17)	O3—N2—C20—C19	6.9 (2)
C11—C1—C10—C5	178.82 (10)	O4—N2—C20—C21	8.0 (2)
C4—C5—C10—C9	−177.95 (11)	O3—N2—C20—C21	−172.45 (15)
C6—C5—C10—C9	1.88 (18)	C19—C20—C21—C22	−1.5 (2)
C4—C5—C10—C1	2.47 (17)	N2—C20—C21—C22	177.76 (12)
C6—C5—C10—C1	−177.70 (11)	C20—C21—C22—C23	0.8 (2)
C18—N1—C11—C12	−173.57 (9)	C21—C22—C23—C18	0.7 (2)
C18—N1—C11—C1	7.81 (16)	C19—C18—C23—C22	−1.47 (18)
C2—C1—C11—N1	−99.60 (14)	N1—C18—C23—C22	−175.47 (11)

### Hydrogen-bond geometry (Å, °)

**Table:**

*D*—H···*A*	*D*—H	H···*A*	*D*···*A*	*D*—H···*A*
O1—H1···O3^i^	0.83 (2)	2.05 (2)	2.8559 (17)	163 (18)
C19—H19···O1	0.95	2.56	3.3241 (16)	138

Symmetry codes: (i) −*x*+1, −*y*, −*z*+2.
